# Supplementary material for: Update Disturbance‐Resilient Analog ReRAM Crossbar Arrays for In‐Memory Deep Learning Accelerators
Source: Adv Sci (Weinh). 2025 Sep 16;13(4):e04578. doi: 10.1002/advs.202504578 (PMC12822454; doi:10.1002/advs.202504578)
Supplement: Supplementary file 1 — Supporting Information [file ADVS-13-e04578-s001.pdf]

Supporting Information

Update Disturbance-Resilient Analog ReRAM Crossbar Arrays for In-Memory Deep Learning Accelerators

Wooseok Choi[1*] Tommaso Stecconi[1*] Donato Francesco Falcone[1] Matteo Galetta[1] Victoria Clerico[1] Elisa Zaccaria[1] Mamidala Saketh Ram[1] Antonio La Porta[1] Folkert Horst[1] Daniel Jubin[1] Matias Senger[1] Marilyne Sousa[1] Steffen Reidt[1] Ralph Heller[1] Bernabe Linares-Barranco[2] Valeria Bragaglia[1] Bert Jan Offrein[1]

{1}IBM Research Europe-Zurich, 8803 Rüschlikon, Switzerland

{2}Instituto de Microelectrónica de Sevilla (IMSE-CNM), CSIC and Univ. de Sevilla, 41092 Sevilla, Spain

E-mail: wooseok.choi@ibm.com

Contents

A. Supplementary Figures 2

B. Supplementary Table 8

C. Supplementary Note 1 8

D. Supplementary Reference 9

A. Supplementary Figures

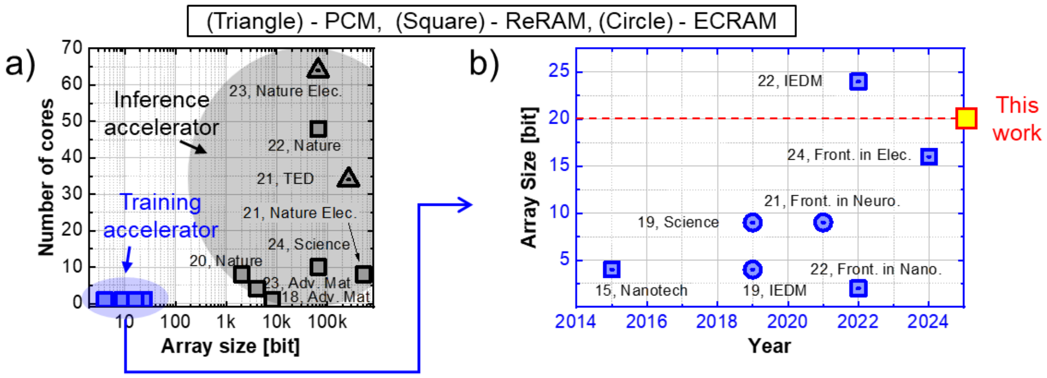

Supplementary Figure 1. (a) The literature on in-memory AI accelerator chips using analog emerging memory devices. Each symbol represents a different device technology, as noted in the legend above the figures. In (a), in-memory inference accelerators have been pre-dominantly demonstrated with a scaled array size and multi-cores in the chip. In contrast, studies on in-memory training accelerators are still in the embryonic stages, as indicated by the blue shaded area. **(b)** The literature on in-memory training works, which demonstrated neural network (NN) in-memory learning. ***Compared to other studies, this work, based on a 20-bit array (5x4), delves into update disturbances in analog memories, which is crucial for scaling up the array size in training accelerators.*** References can be found in the reference list of the main manuscript.

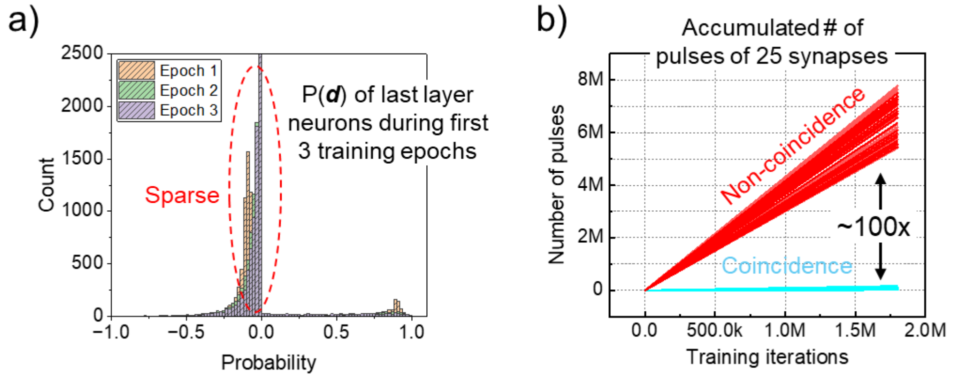

Supplementary Figure 2. We verify the significance of update-disturbance resilience in analog memory devices by estimating the number of non-coincident/coincident pulses during NN training using our established simulator described in the manuscript. **(a)** Histogram of probability-encoded neuron signals, P($\boldsymbol{d}$), from the last neuron layer, where $\boldsymbol{d}$ represents an error vector. We observe that the probability (e.g., P($\boldsymbol{x}$) and P($\boldsymbol{d}$)) are sparse, concentrated around zero, and become even sparser as the training progresses. This sparsity also results in sparse stochastic pulses in the bitstreams for array parallel updates. Therefore, the frequency of pulse non-coincident cases bacomes high. **(b)** The number of pulses experienced by 25 representative synapses as training iterations increase. As expected, the non-coincident pulse cases occur more than the coincident pulse cases more than 100 times. In the stochastic parallel updates, a bitstream length (BL) of 10 was used for this estimation. All NN simulations were conducted under the same conditions as described in the Section 3.4 of the manuscript.

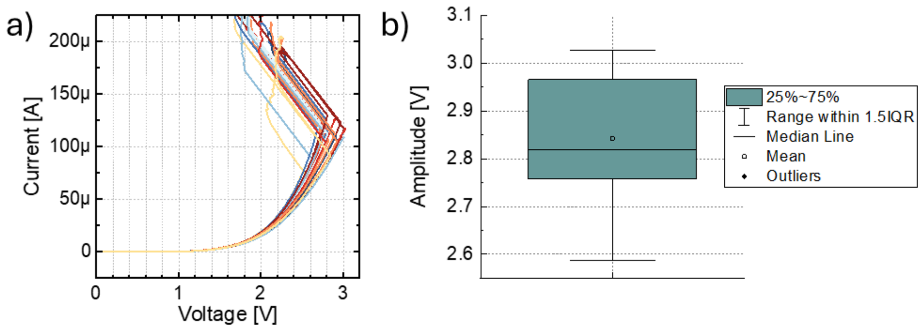

Supplementary Figure 3. (a) Conductive filament formation curves of 20 ReRAM cells. **(b)** A box plot demonstrating an average forming voltage as low as 2.85 V, which is crucial for integration with advanced silicon technology node [1].

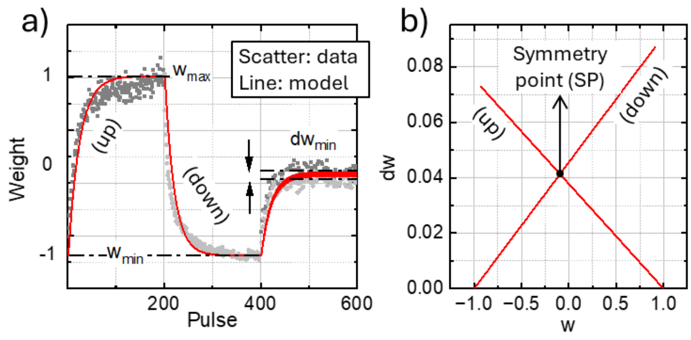

Parameter	Value
W_{min}	-1
W_{max}	1
dw_{min}	0.045
σ±	-0.1

Supplementary Figure 4. (a) Softbound model fitting to the experimental data shown in Figure 2e of the manuscript. **(b)** A line plot showing the amount of weight change per pulse, *dw*, as a function of the current weight value, *w*. The fitting results are shown in the table on the right. The cross-section represents the balanced symmetry point (SP), where the conductance changes per applied up/down pulses are the same. By applying one-up and one-down pulses repetitively, the device finds its SP. The softbound model and fitting methods can also be found in the work by Rasch et al. [2].

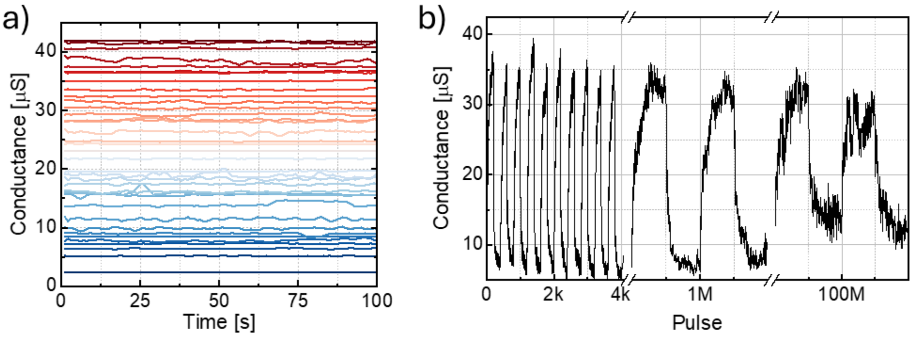

Supplementary Figure 5. (a) 100-second retention test of 32 nonvolatile states during open-loop update operations. The measurements were conducted under a constant voltage stress of 0.2 V after applying programming pulses to the device. Although the device exhibits conductance fluctuations due to metastable oxygen ions under voltage stress, our ReRAM technology demonstrates nonvolatile analog switching. **(b)** For training applications, high cycling endurance is highly desired. The extreme-endurance tests, with more than 100 million programming pulses, validate the analog switching capability of our CMO/HfO_x ReRAM device. As reported in a previous study by Zhao et al. [3], the device shows a reduced conductance window and increased variability of resistive switching.

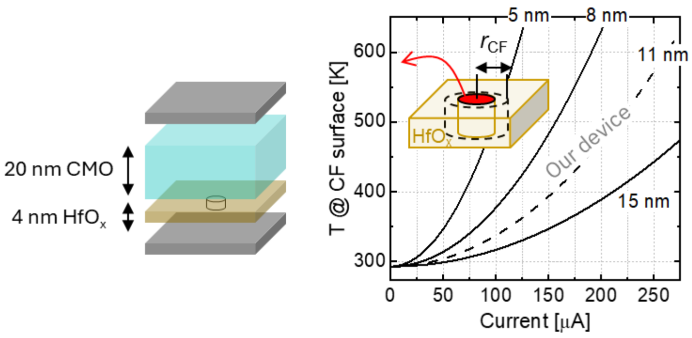

Supplementary Figure 6. The localized resistive switching activation, based on a rigid nanoscale conductive filament (CF), exponentially enhances the Joule heating effects. Through COMSOL Multiphysics simulations, we also investigate the impact of CF dimensions on internal temperature increases. The graph shows the internal temperature at the CF surface as a function of current flow. The results imply that a further-scaled CF can more effectively increase Joule heating, potentially enhancing the switching non-linearity with respect to the applied voltage amplitude.

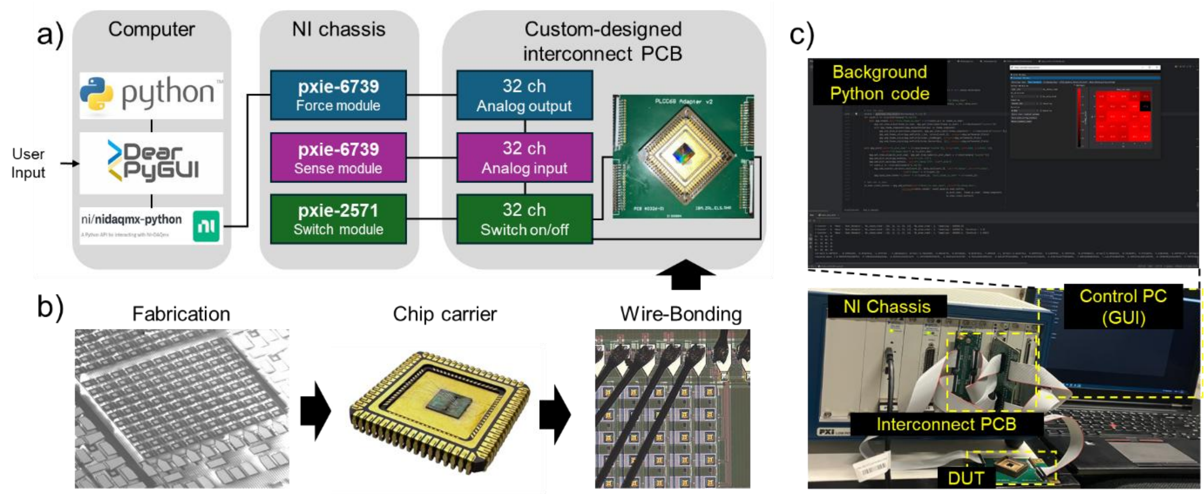

Supplementary Figure 7. (a) The configuration of our array controller setup, based on a National Instruments (NI) machine. We developed a Python-based graphical user interface (GUI) to control the NI system with PXIe cards, which include multi-channel analog input/output (AI/AO) and a relay module. Additionally, we fabricated custom-designed printed circuit boards (PCBs) for interconnections between the PXIe modules. The voltage signals in the individual channels are generated by the AO force module, and applied to the chip passing by the Sense and Switch module. This setup also allows for voltage sensing on the 100-ohm sense resistor in each channel simultaneously. The sample preparation process is shown in **(b)**. **(c)** shows the Python-based GUI environment on the computer and a photo of the array controller setup.

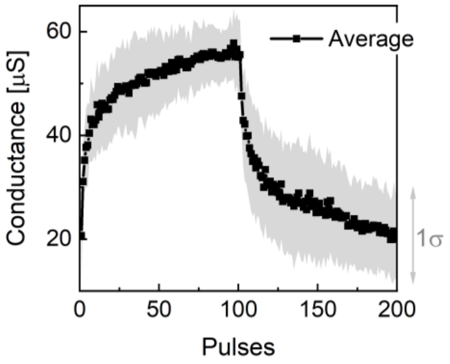

Supplementary Figure 8. Average conductance values (symbols) with one-sigma error bars from 100-pulse cycling experiments conducted on multiple devices across the array. The plot includes the experiemtal data shown in the Figure 4b in the manuscript.

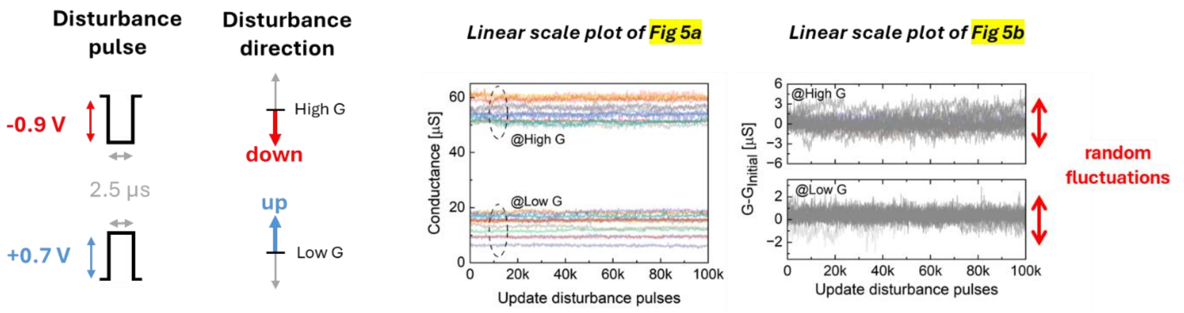

Supplementary Figure 9. Left displays the polarity of the pulses which is chosen to drive the device conductance (G) away from high-G and low-G set values. In the linear-scale plot of Fig.5a, the device G does not show any tendency to drift under this harsh condition of applying 100k disturbance pulses. The linear-scale plot of Fig.5b shows that the device G exhibits random fluctuations in response to the disturbance pulses, which is attributed to the movement of metastable oxygen ions in the device.

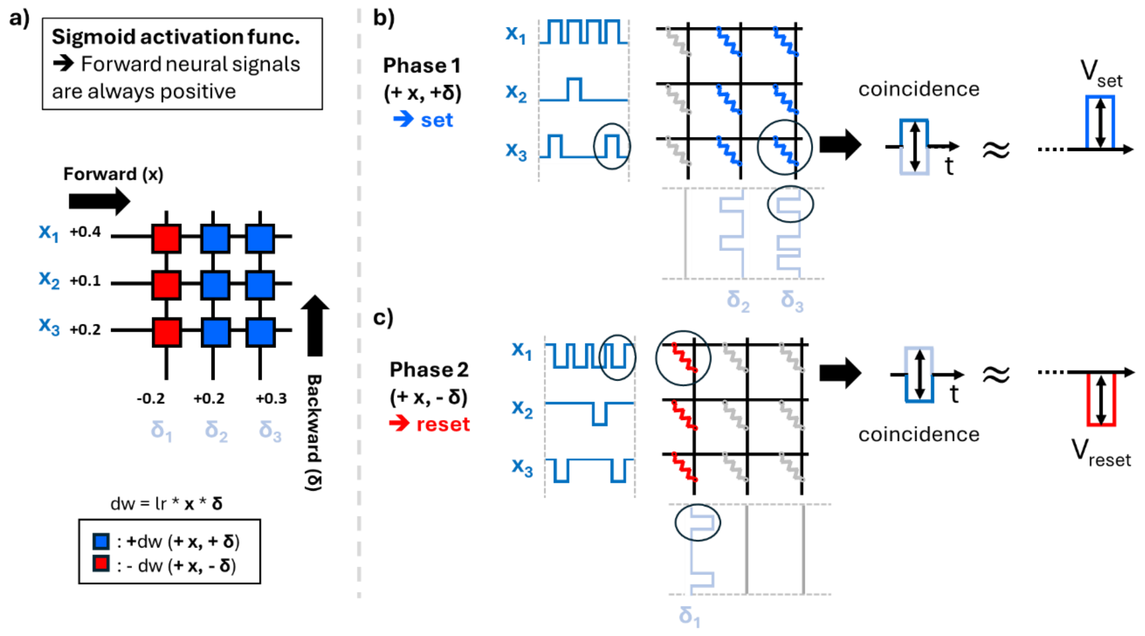

Supplementary Figure 10. Illustrations show the process of stochastic parallel weight updates within each iteration. The fully parallel weight updates consist of sub-phases (two phases in this case with sigmoidal neurons). The parallel weight programming for set and reset operations cannot be performed in parallel at the same time, as the polarities of the applied pulses need to be reversed for set and reset operations. **(a)** shows two different cells that need to be set (blue) and reset (red). **(b) and (c)** display the first and second phases for the parallel set and reset weight updates, respectively. In case of the parallel set operations, the pulse amplitudes on the rows and columns should be +½V_{set} and -½V_{set}, respectively, to have full switching voltage of V_{set}. The number of sub-phases becomes 4 when the neuron activation is based on the tanh function [4].

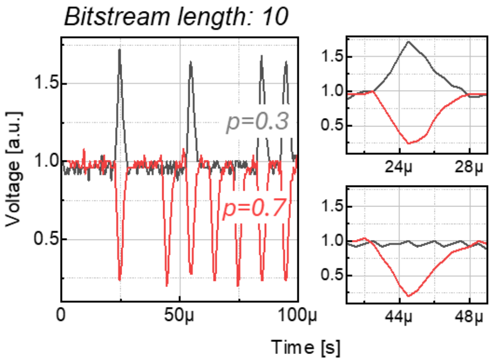

Supplementary Figure 11. Experimentally generated stochastic pulse trains with different probabilities that create pulse (non-)coincident cases. The right figures show examples of the timing for coincident and non-coincident pulses, respectively.

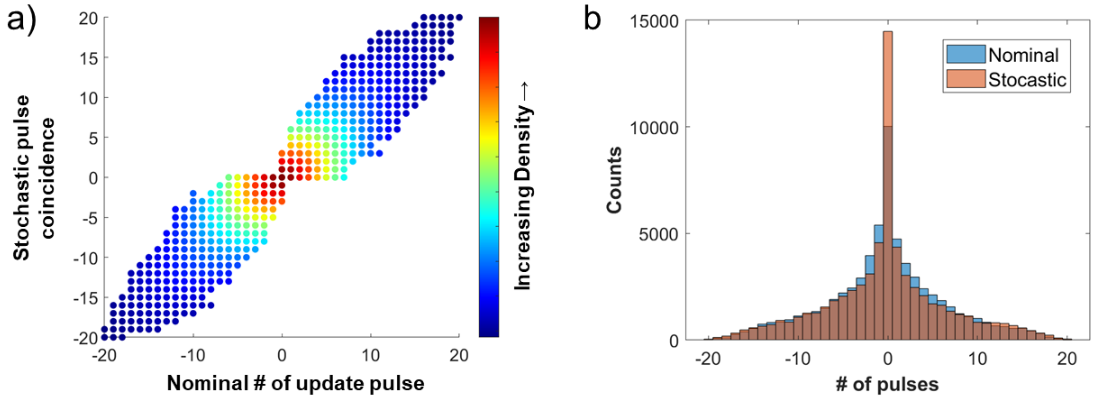

Supplementary Figure 12. The verification of the stochastic fully parallel weight update scheme in a crossbar array. **(a)** The density scatter plot demonstrates the statistical correlation between the desired number of updates in a weight matrix and the resulting number of stochastic pulse coincidence in a ReRAM array. Arbitrary values for the probabilities of ***x*** and ***d*** were used in this simulation experiements, with a stochastic bitstream length (BL) set to 20. The stochastic update scheme also enhances the robustness of the algorithm without the need for an additional stochastic rounding process [4]. **(b)** The histogram of the number of update pulses also shows a close correlation between the nominal update pulses and the stochastic coincidence update pulses.

B. Supplementary Table

Table S1. Potential materials for the CMO layer of CMO/HfO_x ReRAM technology

Material	Ref.	Process	Conductivity [Sm^{-1}]
TaO_x	[5]	Sputtering	$10^{-3} \sim 10^5$
TiO_x	[6]	Sputtering/PLD	$10^{-1} \sim 10^5$
WO_x	[7]	Sputtering	$10^{-5} \sim 10^5$
GaO_x	[8]	PLD	$10^{-2} \sim 10^5$
$VCrO_x$	[9]	Temperature treatment	$10^0 \sim 10^5$

C. Supplementary Note 1

3D Finite Element Model (FEM)

Electrothermal 3D simulations of the CMO/HfO_x ReRAM device are performed using COMSOL Multiphysics. Based on the device configuration after electroforming of the conductive filament (CF), the charge continuity Equation (a) and the Joule-heating Equation (b) are numerically solved to reproduce the experimental IV response and compute the 3D distributions of the electrostatic field ($\mathcal{E}$) and the temperature (T), respectively.

$$\text{(a)} \quad \nabla \cdot J_e = \nabla \cdot \big(\sigma(-\nabla \mathrm{V})\big) = 0$$
$$\text{(b)} \quad \nabla \cdot (-k\nabla T) = J_e \cdot \mathcal{E} = Q_e$$

In (a), J_e is the current density, σ the electrical conductivity and V the electrostatic potential. While in (b), the thermal conductivity is indicated by k, T is the temperature, $\mathcal{E}$ is the electric field and Q_e the heat generated per unit volume due to Joule heating. According to the simulated electric field and temperature distributions in the device material stack, the average values of $\mathcal{E}$ and T are computed.

Device Switching Non-Linearity

The increase of Joule heat (***T***) provides thermal energy to the oxygen ions, enabling them to overcome migration energy barriers and increasing the switching rate significantly. Also, the applied voltage (***V***) alters the potential landscape, further enhancing the ion migration rate within the CMO film [10]. The ion migration rate follows an exponential relationship, as described below:

$$\text{(c)} \quad Rate = Ae^{-\frac{E_A}{k_B T}},$$
$$\text{(d)} \quad E_A = E_{A0} - \alpha qV$$

where αqV is a barrier lowering term due to the applied voltage, $k_B T$ is the thermal energy, and E_{A0} is the energy barrier of the material at zero external voltage.

D. Supplementary Reference

1. Kim, Y., Seo, S.C., Consiglio, S., Jamison, P., Higuchi, H., Rasch, M., Wu, E.Y., Kong, D., Saraf, I., Catano, C. and Muralidhar, R., 2021. Resistive memory process optimization for high resistance switching toward scalable analog compute technology for deep learning. *IEEE Electron Device Letters*, *42*(5), pp.759-762.
2. Rasch, M.J., Carta, F., Fagbohungbe, O. and Gokmen, T., 2024. Fast and robust analog in-memory deep neural network training. *Nature Communications*, *15*(1), p.7133.
3. Zhao, M., Wu, H., Gao, B., Sun, X., Liu, Y., Yao, P., Xi, Y., Li, X., Zhang, Q., Wang, K. and Yu, S., 2018, December. Characterizing endurance degradation of incremental switching in analog RRAM for neuromorphic systems. In *2018 IEEE International Electron Devices Meeting (IEDM)* (pp. 20-2). IEEE.
4. Haensch, W., Gokmen, T. and Puri, R., 2018. The next generation of deep learning hardware: Analog computing. *Proceedings of the IEEE*, *107*(1), pp.108-122.
5. Bao, K., Meng, J., Poplawsky, J.D. and Skowronski, M., 2023. Electrical conductivity of TaOx as function of composition and temperature. *Journal of Non-Crystalline Solids*, *617*, p.122495.
6. Leichtweiss, T., Henning, R.A., Koettgen, J., Schmidt, R.M., Holländer, B., Martin, M., Wuttig, M. and Janek, J., 2014. Amorphous and highly nonstoichiometric titania (TiO x) thin films close to metal-like conductivity. *Journal of Materials Chemistry A*, *2*(18), pp.6631-6640.
7. Choi, W., Kwak, M., Heo, S., Lee, K., Lee, S. and Hwang, H., 2021, December. Hardware neural network using hybrid synapses via transfer learning: WOx nano-resistors and TiOx RRAM synapse for energy-efficient edge-AI sensor. In *2021 IEEE International Electron Devices Meeting (IEDM)* (pp. 23-1). IEEE.
8. Nagarajan, L., De Souza, R.A., Samuelis, D., Valov, I., Börger, A., Janek, J., Becker, K.D., Schmidt, P.C. and Martin, M., 2008. A chemically driven insulator–metal transition in non-stoichiometric and amorphous gallium oxide. *Nature materials*, *7*(5), pp.391-398.
9. McWhan, D.B. and Remeika, J.P., 1970. Metal-insulator transition in (V 1− x Cr x) 2 O 3. *Physical Review B*, *2*(9), p.3734.
10. Koo, Y., Ambrogio, S., Woo, J., Song, J., Ielmini, D. and Hwang, H., 2015. Accelerated retention test method by controlling ion migration barrier of resistive random access memory. *IEEE Electron Device Letters*, *36*(3), pp.238-240.
